# Development and validation of a CT-based body composition model for predicting adverse outcomes in small bowel obstruction

**DOI:** 10.3389/fmed.2026.1807639

**Published:** 2026-06-01

**Authors:** Yanan Shi, Zhendong Wang, Xiaojuan Tian, Feng Wu, Xiaole Ma, Kai Jia, Jiansheng Guo, Tian Yao, He Huang, Yuntong Guo

**Affiliations:** 1 The First Clinical Medical College, Shanxi Medical University, Taiyuan, Shanxi, China; 2 Department of Gastrointestinal Surgery, The First Hospital of Shanxi Medical University, Taiyuan, Shanxi, China; 3 Department of Imaging, The First Hospital of Shanxi Medical University, Taiyuan, Shanxi, China

**Keywords:** body composition, postoperative complications, prognosis, skeletal muscle density, small bowel obstruction, visceral fat area

## Abstract

**Background:**

Postoperative outcomes of small bowel obstruction (SBO) are unpredictable. Current risk models, based on basic clinical parameters, remain imprecise. While body composition abnormalities such as sarcopenia and visceral obesity are known to affect surgical outcomes, a comprehensive model that integrates muscle quality, systemic inflammation, nutrition, and intraoperative factors is lacking.

**Methods:**

We conducted a retrospective cohort study of 270 patients diagnosed with SBO from the First Hospital of Shanxi Medical University between January 2018 and December 2024. Body composition parameters were assessed using computed tomography (CT) at the level of the third lumbar vertebra. Univariate and multivariate logistic regression analyses were performed to identify risk factors for adverse outcomes, including postoperative sepsis, ICU admission, and postoperative complications.

**Results:**

Of the 270 patients included in the final analysis, 68 required ICU admission, 105 experienced clinically significant postoperative complications (Clavien-Dindo grade ≥ II), and 53 developed postoperative sepsis. Multivariable analysis identified low skeletal muscle density (SMD) as the most consistent and potent independent predictor across all adverse outcomes: postoperative sepsis (aOR 4.58, 95% CI 1.61–13.04, *p* = 0.004), ICU admission (aOR 3.76, 95% CI 1.55–9.13, *p* = 0.003), and postoperative complications (aOR 3.73, 95% CI 1.59–8.71, *p* = 0.002). Other significant predictors included a prolonged time from symptom onset to surgery, longer operative duration, lower serum albumin levels, elevated D-dimer, greater resected bowel length, and prolonged prothrombin time. The predictive models demonstrated strong discriminative ability, with area under the curve (AUC) values ranging from 0.78 to 0.84 in the training sets and 0.72 to 0.80 in the validation sets. Calibration and decision curve analyses confirmed excellent model fit and clinical utility.

**Conclusion:**

We developed and validated a novel multidimensional nomogram that accurately predicts the risk of major postoperative adverse events in SBO patients. By leveraging routinely available end-of-surgery data, this tool facilitates early postoperative risk stratification, which is crucial for intensifying postoperative monitoring and optimizing ICU resource allocation.

## 1 Introduction

Small bowel obstruction (SBO) represents a prevalent surgical emergency with diverse etiologies, including postoperative adhesions, hernias, and neoplasms (1–3). While conservative management suffices for most patients, approximately 20 to 30% require surgical intervention, a proportion consistently observed in a US population-based study and other international cohorts (4, 5). The timing of surgery is critical, as delays are strongly correlated with severe complications such as intestinal ischemia, perforation, and postoperative sepsis, leading to prolonged hospitalization and worsened outcomes (6, 7). Current risk assessment relies on clinical presentation, laboratory markers, and CT imaging features, yet their predictive accuracy for major complications remains suboptimal (8, 9). Furthermore, existing surgical risk models, predominantly derived from elective surgery populations, demonstrate limited applicability in the acute SBO setting, highlighting the need for a dedicated predictive tool (10).

Beyond conventional parameters, there is growing emphasis on body composition and systemic inflammation as determinants of surgical outcomes (11–13). CT-based analysis at the third lumbar vertebra allows for precise quantification of skeletal muscle and adipose tissue, providing objective metrics of nutritional and functional status that surpass traditional anthropometry (14). Concurrently, the pathophysiology of SBO involves intestinal barrier disruption and bacterial translocation, triggering a systemic inflammatory response (15). Composite indices such as the neutrophil-to-lymphocyte ratio (NLR) and platelet-to-lymphocyte ratio (PLR) offer a holistic view of this inflammatory state, while nutritional markers like serum albumin (ALB) reflect the patient’s metabolic reserve (16, 17). However, current research often examines these factors—body composition, inflammation, and nutrition—in isolation, failing to capture their synergistic predictive potential.

Moreover, a critical gap in most prediction models is the omission of intraoperative factors. The operation itself constitutes a major physiological insult, and variables such as surgical duration, extent of bowel resection, and the presence of necrosis dynamically alter the patient’s risk profile (18, 19). Ignoring these factors limits the models’ real-world utility for early postoperative risk stratification.

To address these comprehensive limitations, this study seeks to develop and validate a novel multidimensional predictive model. By integrating body composition parameters, systemic inflammatory-nutritional indices, and key intraoperative variables, we aim to construct and validate a practical nomogram for accurately predicting the risk of clinically significant postoperative complications, postoperative sepsis, and ICU admission in SBO patients. We hypothesize that such a model will outperform existing clinical assessments and provide a robust tool for personalized postoperative management.

## 2 Materials and methods

### 2.1 Study design and patient population

This single-center, retrospective cohort study was approved by the Institutional Review Board of The First Hospital of Shanxi Medical University with a waiver for informed consent. We consecutively screened all adult patients (≥18 years) who were admitted and underwent surgical intervention for a diagnosis of small bowel obstruction (SBO) between January 2018 and December 2024.

The inclusion criteria were: (1) Consecutive adult patients (aged ≥ 18 years) who underwent emergency surgery for a confirmed diagnosis of small bowel obstruction; (2) Availability of an abdominal computed tomography (CT) scan performed at our institution within 48 h prior to the surgical intervention; (3) Complete and accessible medical records for data extraction, including detailed operative notes and postoperative outcomes. Key exclusion criteria included: (1) SBO caused by malignant tumors; (2) presence of severe underlying comorbidities; (3) poor-quality CT images precluding accurate body composition analysis.

### 2.2 Data collection and variables

Clinical data were meticulously extracted from the electronic medical record system. The collected variables primarily included: (1) Demographic characteristics: age, sex, body mass index (BMI), and medical history (hypertension, heart disease, diabetes, brain disease; each as yes/no). (2) Preoperative laboratory parameters: complete blood count (white blood cell count [WBC], red blood cell count [RBC], hemoglobin [Hb], platelet count [Plt]); inflammatory indices, including the neutrophil-to-lymphocyte ratio (NLR), platelet-to-lymphocyte ratio (PLR), and systemic immune-inflammation index (SII, calculated as platelet count × neutrophil count / lymphocyte count); coagulation profiles (D-dimer [mg/L], fibrinogen [FIB, g/L], prothrombin time [PT, seconds]); and nutritional markers (serum albumin [ALB, g/L] and prognostic nutritional index [PNI, calculated as ALB (g/L) + 5 × lymphocyte count (×10^9^/L)]). All laboratory parameters and CT scans were obtained within 48 h before surgery, and the time point closest to surgery was used for analysis. (3) Operative details: presence of small intestinal necrosis (yes/no), resected bowel length (meters), and duration of surgery (minutes). Time from onset to surgery was defined as the interval in days from patient-reported symptom onset, including abdominal pain, distension, vomiting, or obstipation, to surgical incision, encompassing both pre-hospital and in-hospital delays. The primary outcomes were: postoperative sepsis according to Sepsis-3 criteria (20), unplanned intensive care unit (ICU) admission, and clinically significant postoperative complications defined as Clavien-Dindo grade ≥ II (any deviation from normal postoperative course requiring pharmacological treatment or beyond) (21). All postoperative outcomes were assessed within 30 days after surgery.

### 2.3 Measurement of body composition parameters

Body composition analysis was performed on preoperative abdominal CT scans at the level of the third lumbar vertebra (L3). A primary researcher, blinded to all patient outcomes, conducted all measurements using the sliceOmatic image processing system (version 5.0, TomoVision, Montreal, Canada). To assess inter-rater reliability, a second investigator, also blinded to outcomes, independently re-measured body composition parameters in a randomly selected subset of 60 patients (22% of the cohort). The intraclass correlation coefficient for skeletal muscle density (SMD) was 0.95 (95% CI 0.91–0.97), with similarly high reliability for other compartments (Supplementary Table S1). Skeletal muscle was quantified using a Hounsfield Unit (HU) threshold range of −29 to +150, and adipose tissue was segmented into subcutaneous (SFA; −190 to −30 HU) and visceral (VFA; −150 to −50 HU) compartments, according to established protocols (22). The cross-sectional area (cm^2^) of each tissue compartment was automatically computed. Skeletal muscle index (SMI) was calculated as the skeletal muscle area normalized to height squared (cm^2^/m^2^). Skeletal muscle density (SMD) was measured as the mean radiation attenuation (HU) of the same muscle region. The visceral-to-subcutaneous fat ratio (VSR) was subsequently computed. All these indices were dichotomized into ‘low’ and ‘high’ groups based on sex-specific median values (23).

### 2.4 Statistical analysis

Statistical analyses were performed using R software. Complete-case analysis was performed, as there were no missing data for any of the variables included in the final models. This was ensured by the inclusion criteria requiring complete medical records, accessible operative notes, and CT images of sufficient quality for body composition analysis. The total cohort of 270 patients was randomly divided into a training set (70%, *n* = 189) and a validation set (30%, *n* = 81). Continuous variables were compared using Student’s t-test or Mann–Whitney U test, as appropriate, while categorical variables were compared using the Chi-square test or Fisher’s exact test. All univariate and multivariable analyses were conducted in the training set. Multivariable logistic regression analysis was performed using a two-step variable selection procedure within the training set. First, all variables with a univariate association of *p* < 0.05 were entered into the initial full model. Second, a backward stepwise elimination method based on the Akaike Information Criterion (AIC) was applied. Variables that remained after the elimination process were retained in the final model regardless of their individual *p*-values, as they either contributed to model performance or had clear clinical significance. A nomogram was then constructed based on the final set of selected variables. The results of the final models are presented as odds ratios (OR) with 95% confidence intervals (CI). Model performance was evaluated using the validation set. Discrimination was assessed by the area under the receiver operating characteristic curve (AUC-ROC). Calibration was evaluated using calibration plots, which were generated with 1,000 bootstrap resamples to estimate calibration slopes and intercepts and to correct for overfitting in calibration performance. Clinical utility was assessed by decision curve analysis (DCA) (24, 25). All performance metrics were evaluated in both the training and validation sets. A two-tailed *p*-value < 0.05 was considered statistically significant for univariate comparisons and for the initial screening step; the final model retention did not rely on a p-value threshold. Multicollinearity among the variables retained in the final models was assessed using Pearson correlation coefficients (|r| > 0.7 considered indicative of potential collinearity) and variance inflation factor (VIF, with VIF > 5 considered problematic). The highest absolute correlation among final model variables was 0.35, and all VIF values were ≤ 1.14, confirming the absence of significant multicollinearity.

## 3 Results

### 3.1 Patient

This retrospective cohort study screened 354 consecutive patients who underwent surgical management for small bowel obstruction at the First Hospital of Shanxi Medical University over a seven-year period, spanning from January 2018 to December 2024. Following the application of exclusion criteria, a final cohort of 270 patients was deemed eligible for analysis.

**Figure 1:**
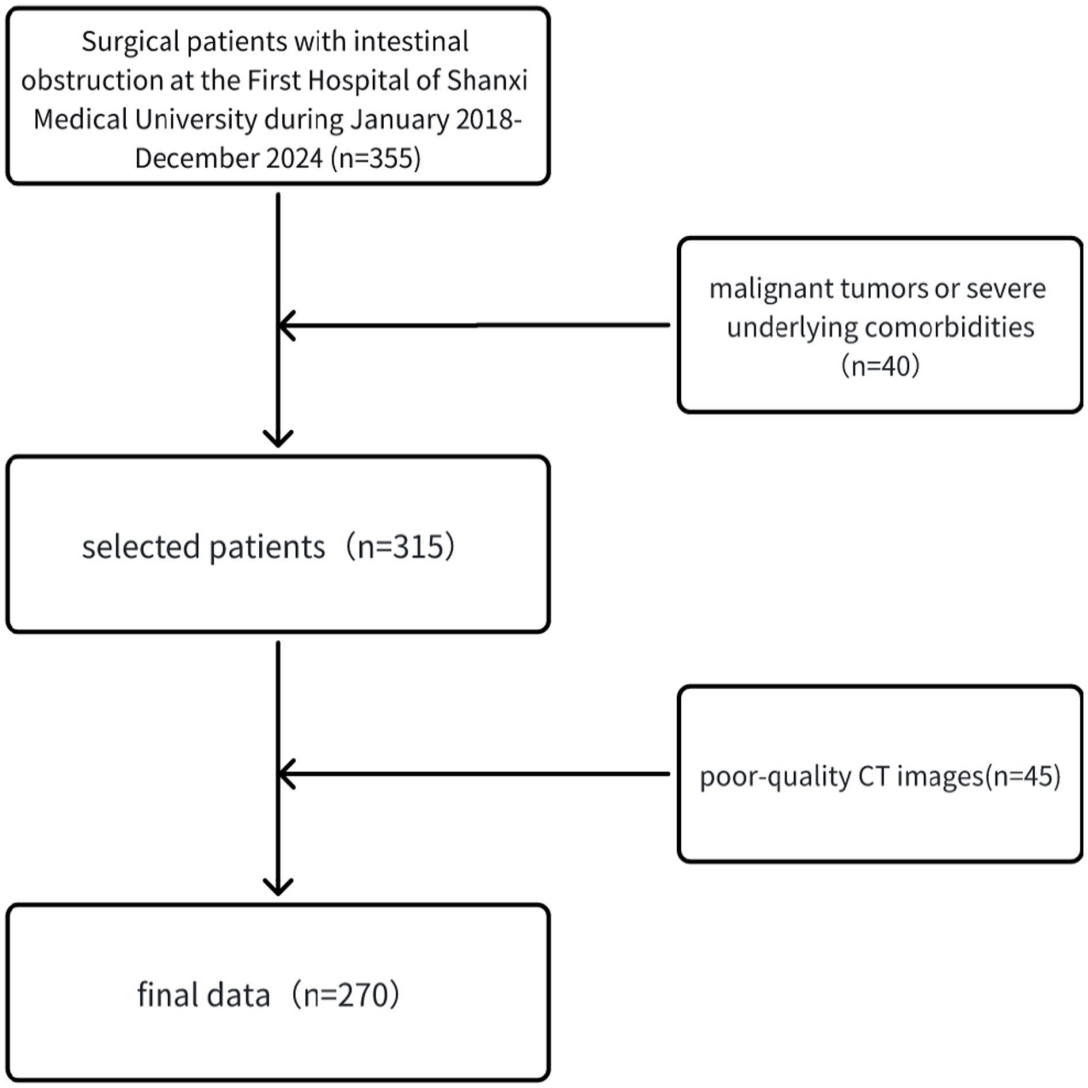
Participant selection flowchart.

During the postoperative period, 68 patients required admission to the intensive care unit (ICU). Clinically significant postoperative complications (Clavien-Dindo grade ≥ II) were documented in 105 individuals, and postoperative sepsis occurred in 53 patients. A detailed flowchart outlining patient selection is provided in Figure 1. Analysis of outcome incidence across the study period showed no significant temporal variation (Supplementary Table S2). The demographic and clinical characteristics of the training and validation cohorts were well balanced, as shown in Supplementary Table S3.

### 3.2 Predictors of postoperative sepsis

The baseline characteristics of patients with and without postoperative sepsis are compared in Table 1, while the corresponding univariate and multivariate logistic regression analyses are summarized in Table 2. Univariate analysis identified several factors significantly associated with the development of postoperative sepsis. These included advanced age (OR 1.04, 95% CI 1.01–1.07, *p* = 0.004), pre-existing brain disease (OR 4.26, 95% CI 1.17–15.56, *p* = 0.028), longer time from onset to surgery (OR 1.11, 95% CI 1.04–1.18, *p* = 0.003), low SMI (OR 2.41, 95% CI 1.15–5.05, *p* = 0.020), and low SMD (OR 4.95, 95% CI 2.06–11.92, *p* < 0.001). Among body composition parameters, low visceral fat area (VFA) was associated with a reduced risk (OR 0.44, 95% CI 0.21–0.92, *p* = 0.028). Significant laboratory predictors were lower albumin levels (ALB; OR 0.88, 95% CI 0.83–0.93, *p* < 0.001) and elevated D-dimer (OR 1.57, 95% CI 1.23–1.99, *p* < 0.001). Univariate associations of body composition parameters as continuous variables are provided in Supplementary Table S4 for dose–response evaluation.

**Table 1:** Comparison of clinical characteristics between patients with and without postoperative sepsis.

Variables	Postoperative sepsis (*n* = 53)	No postoperative sepsis (*n* = 217)	Statistic	*p*-value
Age, Mean ± SD	69.57 ± 13.35	61.21 ± 15.50	t = 3.61	<0.001
Sex, n(%)			χ^2^ = 0.01	0.926
Female	26 (49.06)	108 (49.77)		
Male	27 (50.94)	109 (50.23)		
BMI, Mean ± SD	22.27 ± 5.31	21.68 ± 3.98	t = 0.91	0.366
Hypertension, n(%)			χ^2^ = 0.09	0.764
No	38 (71.70)	160 (73.73)		
Yes	15 (28.30)	57 (26.27)		
Heart disease, n(%)			χ^2^ = 1.19	0.276
No	46 (86.79)	201 (92.63)		
Yes	7 (13.21)	16 (7.37)		
Diabetes, n(%)			χ^2^ = 0.06	0.803
No	48 (90.57)	194 (89.40)		
Yes	5 (9.43)	23 (10.60)		
Brain Disease, n(%)			χ^2^ = 1.46	0.227
No	47 (88.68)	205 (94.47)		
Yes	6 (11.32)	12 (5.53)		
Time from onset to surgery(d), M (Q₁, Q₃)	4.00 (3.00, 9.00)	3.00 (1.00, 5.00)	Z = −3.68	<0.001
Small intestinal necrosis, n(%)			χ^2^ = 4.61	0.032
Yes	43 (81.13)	143 (65.90)		
No	10 (18.87)	74 (34.10)		
Resected bowel length(m), M (Q₁, Q₃)	0.25 (0.10, 0.90)	0.10 (0.00, 0.35)	Z = −2.92	0.003
Duration of surgery(min), M (Q₁, Q₃)	123.00 (100.00, 180.00)	120.00 (90.00, 150.00)	Z = −.09	0.037
SMI, n(%)			χ^2^ = 6.78	0.009
High	18 (33.96)	117 (53.92)		
Low	35 (66.04)	100 (46.08)		
SMD, n(%)			χ^2^ = 25.57	<0.001
High	10 (18.87)	125 (57.60)		
Low	43 (81.13)	92 (42.40)		
SFA, n(%)			χ^2^ = 0.59	0.444
High	29 (54.72)	106 (48.85)		
Low	24 (45.28)	111 (51.15)		
VFA, n(%)			χ^2^ = 8.47	0.004
High	36 (67.92)	99 (45.62)		
Low	17 (32.08)	118 (54.38)		
WBC(×10^9^/L), M (Q₁, Q₃)	9.20 (6.20, 12.90)	9.00 (6.50, 11.90)	Z = −0.27	0.785
RBC(×10^9^/L), M (Q₁, Q₃)	4.35 (3.64, 4.99)	4.40 (4.02, 5.02)	Z = −1.30	0.194
Hb(g/L), M (Q₁, Q₃)	132.00 (110.00, 151.00)	136.00 (123.00, 153.00)	Z = −1.61	0.108
Plt(×10^9^/L), M (Q₁, Q₃)	196.00 (150.00, 273.00)	231.00 (175.00, 288.00)	Z = −1.44	0.151
NLR, M (Q₁, Q₃)	8.92 (4.92, 19.25)	7.11 (4.41, 13.34)	Z = −1.57	0.116
PLR, M (Q₁, Q₃)	289.30 (175.58, 411.11)	232.77 (161.54, 355.00)	Z = −1.33	0.182
SII, M (Q₁, Q₃)	1494.49 (1095.40, 4525.02)	1582.24 (892.57, 3175.93)	Z = −0.71	0.475
PNI, M (Q₁, Q₃)	38.40 (32.25, 41.40)	44.50 (38.75, 48.95)	Z = −4.75	<0.001
ALB(g/L), M (Q₁, Q₃)	31.70 (27.90, 37.20)	38.70 (34.60, 42.80)	Z = −5.10	<0.001
D-dimer(mg/L), M (Q₁, Q₃)	1.60 (0.92, 3.42)	0.53 (0.28, 1.03)	Z = −6.56	<0.001
PT(seconds), M (Q₁, Q₃)	15.60 (14.20, 17.00)	14.60 (13.50, 15.60)	Z = −3.08	0.002
FIB(g/L), M (Q₁, Q₃)	3.90 (2.80, 5.55)	3.34 (2.78, 4.12)	Z = −2.52	0.012
APTT(seconds), M (Q₁, Q₃)	28.80 (27.00, 31.70)	29.60 (27.50, 32.10)	Z = −1.05	0.296

**Table 2:** Univariate and multivariate logistic regression analysis for predictors of postoperative sepsis.

Variables	Univariate analysis					Multivariate analysis				
	β	S. E	Z	*p*	OR (95%CI)	*β*	S. E	Z	*p*	OR (95%CI)
Age	0.04	0.01	2.88	**0.004**	1.04 (1.01–1.07)					
Sex										
Female					1.00 (Reference)					
Male	0.08	0.36	0.22	0.828	1.08 (0.53–2.19)					
BMI	0.01	0.04	0.25	0.805	1.01 (0.93–1.09)					
Hypertension										
No					1.00 (Reference)					
Yes	−0.26	0.42	−0.62	0.538	0.77 (0.34–1.76)					
Heart disease										
No					1.00 (Reference)					
Yes	0.74	0.54	1.38	0.169	2.09 (0.73–5.98)					
Diabetes										
No					1.00 (Reference)					
Yes	−0.04	0.59	−0.07	0.941	0.96 (0.30–3.04)					
Brain disease										
No					1.00 (Reference)					
Yes	1.45	0.66	2.20	**0.028**	4.26 (1.17–15.56)					
Time from onset to surgery(d)	0.10	0.03	3.00	**0.003**	1.11 (1.04–1.18)	0.12	0.04	2.88	**0.004**	1.13 (1.04–1.22)
Small intestinal necrosis										
Yes					1.00 (Reference)					
No	−0.80	0.45	−1.76	0.078	0.45 (0.19–1.09)					
Resected bowel length(m)	0.56	0.31	1.83	0.067	1.75 (0.96–3.18)					
Duration of surgery(min)	0.00	0.00	1.49	0.135	1.00 (1.00–1.01)					
SMI										
High					1.00 (Reference)					
Low	0.88	0.38	2.33	**0.020**	2.41 (1.15–5.05)					
SMD										
High					1.00 (Reference)					1.00 (Reference)
Low	1.60	0.45	3.57	**<0.001**	4.95 (2.06–11.92)	1.52	0.53	2.85	**0.004**	4.58 (1.61–13.04)
SFA										
High					1.00 (Reference)					
Low	−0.23	0.36	−0.65	0.516	0.79 (0.39–1.60)					
VFA										
High					1.00 (Reference)					1.00 (Reference)
Low	−0.83	0.38	−2.19	**0.028**	0.44 (0.21–0.92)	−0.63	0.44	−1.44	0.151	0.53 (0.23–1.26)
VSR										
High					1.00 (Reference)					
Low	−0.07	0.36	−0.21	0.837	0.93 (0.46–1.88)					
WBC(×10^9^/L)	0.01	0.03	0.39	0.696	1.01 (0.96–1.07)					
RBC(×10^9^/L)	−0.28	0.23	−1.22	0.222	0.76 (0.49–1.18)					
Hb(g/L)	−0.01	0.01	−1.66	0.097	0.99 (0.97–1.00)					
Plt(×10^9^/L)	0.00	0.00	0.02	0.981	1.00 (1.00–1.00)					
NLR	0.02	0.02	1.19	0.233	1.02 (0.99–1.06)					
PLR	0.00	0.00	1.32	0.186	1.00 (1.00–1.00)					
SII	0.00	0.00	0.35	0.729	1.00 (1.00–1.00)					
PNI	0.00	0.01	0.01	0.989	1.00 (0.98–1.02)					
ALB(g/L)	−0.13	0.03	−4.37	**<0.001**	0.88 (0.83–0.93)	−0.09	0.04	−2.68	**0.007**	0.91 (0.85–0.97)
D-dimer(mg/L)	0.45	0.12	3.69	**<0.001**	1.57 (1.23–1.99)	0.28	0.12	2.36	**0.018**	1.33 (1.05–1.68)
PT(seconds)	0.11	0.07	1.64	0.100	1.12 (0.98–1.28)					
FIB(g/L)	0.19	0.11	1.74	0.082	1.21 (0.98–1.51)					
APTT(seconds)	−0.01	0.04	−0.34	0.737	0.99 (0.91–1.07)					

OR, Odds Ratio; CI, Confidence Interval. The bold values represent *p* ≤ 0.05.

**Figure 2:**
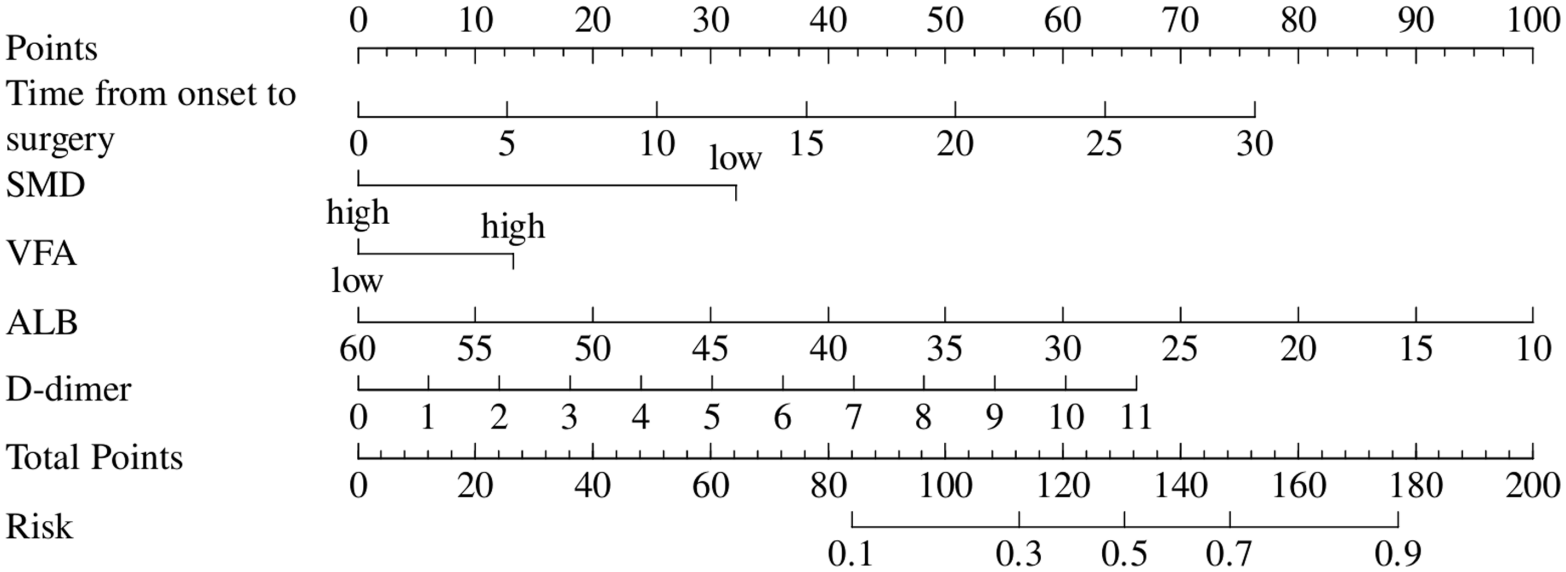
Nomogram for predicting postoperative sepsis.

In the multivariable analysis, four independent predictors for postoperative sepsis were retained. The model confirmed that longer time from onset to surgery (aOR 1.13, 95% CI 1.04–1.22, *p* = 0.004), low SMD (aOR 4.58, 95% CI 1.61–13.04, *p* = 0.004), lower ALB (aOR 0.91, 95% CI 0.85–0.97, *p* = 0.007), and elevated D-dimer (aOR 1.33, 95% CI 1.05–1.68, *p* = 0.018) were independently associated with sepsis risk. Low VFA, which was significant in univariate analysis, was not retained as an independent predictor in the final multivariable model (aOR 0.53, 95% CI 0.23–1.26, *p* = 0.151). A nomogram incorporating these variables was constructed (Figure 2).

**Figure 3:**
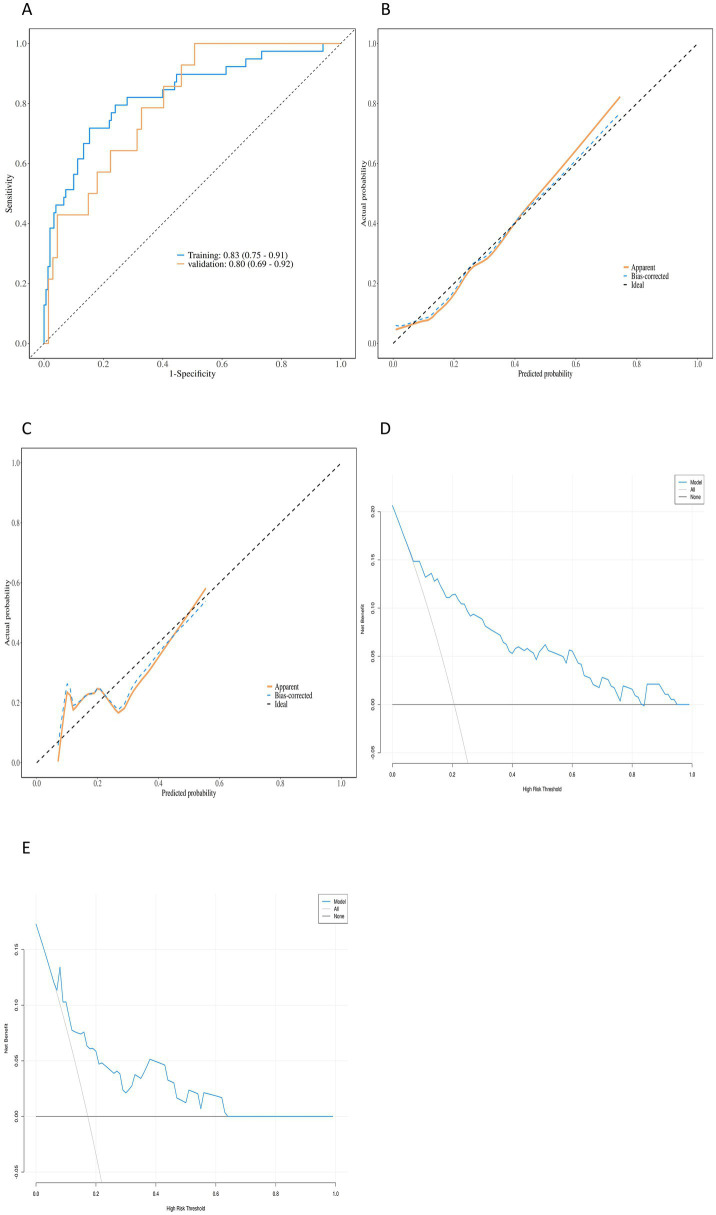
**(A)** ROC curves for the postoperative sepsis prediction model in training and validation sets; **(B)** Calibration curves for the postoperative sepsis prediction model in training sets; **(C)** Calibration curves for the postoperative sepsis prediction model in validation sets; **(D)** Decision curve analysis for the postoperative sepsis prediction model in training sets; **(E)** Decision curve analysis for the postoperative sepsis prediction model in validation sets.

The model demonstrated strong and consistent performance. The AUC was 0.83 (95% CI: 0.75–0.91) in the training set and 0.80 (95% CI: 0.69–0.92) in the validation set (Figure 3A). Calibration curves showed excellent agreement between predictions and observations in both cohorts (Figures 3B,C). Decision curve analysis confirmed the model’s superior clinical utility across a wide range of risk thresholds (Figures 3D,E).

### 3.3 Predictors of ICU admission

Table 3 outlines the clinical characteristics stratified by ICU admission status. The predictive model for ICU admission, constructed via multivariable logistic regression, is displayed in Table 4. Univariate analysis identified several factors significantly associated with ICU admission. Advanced age (OR 1.05, 95% CI 1.02–1.08, *p* < 0.001), longer time from onset to surgery (OR 1.08, 95% CI 1.01–1.15, *p* = 0.023), greater resected bowel length (OR 2.04, 95% CI 1.15–3.62, *p* = 0.015), low SMI (OR 2.89, 95% CI 1.44–5.78, *p* = 0.003), and low SMD (OR 4.32, 95% CI 2.00–9.34, *p* < 0.001) all demonstrated significant positive associations. Laboratory parameters including lower albumin levels (ALB; OR 0.87, 95% CI 0.82–0.92, *p* < 0.001), elevated D-dimer (OR 1.39, 95% CI 1.12–1.73, *p* = 0.003), and prolonged prothrombin time (PT; OR 1.23, 95% CI 1.07–1.40, *p* = 0.003) were also significantly associated with ICU admission. Continuous body composition parameters showed consistent dose–response relationships (Supplementary Table S4).

**Table 3:** Comparison of clinical characteristics between patients with and without ICU admission.

Variables	No ICU (*n* = 202)	ICU (*n* = 68)	Statistic	*p*-value
Age, Mean ± SD	60.63 ± 15.35	69.44 ± 13.84	t = −4.19	<0.001
Sex, n(%)			χ^2^ = 0.04	0.834
Female	101 (50.00)	33 (48.53)		
Male	101 (50.00)	35 (51.47)		
BMI, Mean ± SD	21.79 ± 3.86	21.83 ± 5.33	t = −0.06	0.951
Hypertension, n(%)			χ^2^ = 0.35	0.554
No	150 (74.26)	48 (70.59)		
Yes	52 (25.74)	20 (29.41)		
Heart disease, n(%)			χ^2^ = 4.47	0.035
No	189 (93.56)	58 (85.29)		
Yes	13 (6.44)	10 (14.71)		
Diabetes, n(%)			χ^2^ = 0.23	0.629
No	180 (89.11)	62 (91.18)		
Yes	22 (10.89)	6 (8.82)		
Brain Disease, n(%)			χ^2^ = 1.22	0.269
No	191 (94.55)	61 (89.71)		
Yes	11 (5.45)	7 (10.29)		
Time from onset to surgery(d), M (Q₁, Q₃)	3.00 (1.00, 5.00)	4.00 (2.00, 8.00)	Z = −2.78	0.005
Small intestinal necrosis, n(%)			χ^2^ = 4.70	0.030
Yes	132 (65.35)	54 (79.41)		
No	70 (34.65)	14 (20.59)		
Resected bowel length(m), M (Q₁, Q₃)	0.10 (0.00, 0.30)	0.28 (0.09, 0.93)	Z = −3.52	<0.001
Duration of surgery(min), M (Q₁, Q₃)	120.00 (90.00, 146.75)	123.50 (100.00, 181.00)	Z = −2.51	0.012
SMI, n(%)			χ^2^ = 13.29	<0.001
High	114 (56.44)	21 (30.88)		
Low	88 (43.56)	47 (69.12)		
SMD, n(%)			χ^2^ = 22.72	<0.001
High	118 (58.42)	17 (25.00)		
Low	84 (41.58)	51 (75.00)		
SFA, n(%)			χ^2^ = 0.00	1.000
High	101 (50.00)	34 (50.00)		
Low	101 (50.00)	34 (50.00)		
VFA, n(%)			χ^2^ = 3.85	0.050
High	94 (46.53)	41 (60.29)		
Low	108 (53.47)	27 (39.71)		
WBC(×10^9^/L), M (Q₁, Q₃)	8.80 (6.35, 11.88)	9.25 (6.38, 12.83)	Z = −0.65	0.519
RBC(×10^9^/L), M (Q₁, Q₃)	4.42 (4.03, 5.02)	4.23 (3.68, 4.96)	Z = −1.94	0.053
Hb(g/L), M (Q₁, Q₃)	137.50 (124.00, 153.00)	132.00 (114.50, 146.25)	Z = −1.82	0.068
Plt(×10^9^/L), M (Q₁, Q₃)	232.00 (176.00, 290.25)	207.00 (142.75, 279.50)	Z = −1.78	0.076
NLR, M (Q₁, Q₃)	7.05 (4.34, 13.96)	8.43 (4.99, 15.82)	Z = −1.52	0.129
PLR, M (Q₁, Q₃)	242.78 (161.85, 360.86)	247.78 (171.46, 403.28)	Z = −0.55	0.581
SII, M (Q₁, Q₃)	1581.88 (882.68, 3205.87)	1520.13 (1085.21, 3619.58)	Z = −0.57	0.566
PNI, M (Q₁, Q₃)	44.65 (39.17, 49.34)	38.42 (32.33, 43.31)	Z = −5.25	<0.001
ALB(g/L), M (Q₁, Q₃)	39.05 (35.00, 43.10)	33.45 (27.90, 37.58)	Z = −5.63	<0.001
D-dimer(mg/L), M (Q₁, Q₃)	0.48 (0.28, 1.01)	1.19 (0.73, 2.66)	Z = −6.25	<0.001
PT(seconds), M (Q₁, Q₃)	14.60 (13.50, 15.50)	15.55 (14.10, 17.20)	Z = −3.31	<0.001
FIB(g/L), M (Q₁, Q₃)	3.35 (2.78, 4.12)	3.75 (2.81, 4.83)	Z = −1.95	0.052
APTT(seconds), M (Q₁, Q₃)	29.50 (27.42, 32.25)	29.70 (27.40, 31.63)	Z = −0.76	0.446

**Table 4:** Univariate and multivariate logistic regression analysis for predictors of ICU admission.

Variables	Univariate analysis					Multivariate analysis				
	β	S. E	Z	*p*	OR (95%CI)	β	S. E	Z	*p*	OR (95%CI)
Age	0.05	0.01	3.30	**<0.001**	1.05 (1.02–1.08)					
Sex										
Female					1.00 (Reference)					
Male	0.01	0.33	0.04	0.966	1.01 (0.53–1.95)					
BMI	−0.03	0.04	−0.69	0.488	0.97 (0.90–1.05)					
Hypertension										
No					1.00 (Reference)					
Yes	−0.14	0.38	−0.36	0.720	0.87 (0.41–1.85)					
Heart disease										
No					1.00 (Reference)					
Yes	0.96	0.51	1.90	0.058	2.62 (0.97–7.09)					
Diabetes										
No					1.00 (Reference)					
Yes	−0.02	0.55	−0.04	0.966	0.98 (0.34–2.85)					
Brain disease										
No					1.00 (Reference)					
Yes	1.15	0.66	1.75	0.079	3.16 (0.87–11.45)					
Time from onset to surgery(d)	0.07	0.03	2.28	**0.023**	1.08 (1.01–1.15)	0.10	0.04	2.47	**0.014**	1.10 (1.02–1.19)
Small intestinal necrosis										
Yes					1.00 (Reference)					
No	−0.77	0.41	−1.88	0.060	0.46 (0.21–1.03)					
Resected bowel length(m)	0.71	0.29	2.44	**0.015**	2.04 (1.15–3.62)	0.76	0.36	2.12	**0.034**	2.14 (1.06–4.33)
Duration of surgery(min)	0.01	0.00	1.95	0.051	1.01 (1.00–1.01)					
SMI										
High					1.00 (Reference)					
Low	1.06	0.35	2.99	**0.003**	2.89 (1.44–5.78)					
SMD										
High					1.00 (Reference)					1.00 (Reference)
Low	1.46	0.39	3.72	**<0.001**	4.32 (2.00–9.34)	1.33	0.45	2.93	**0.003**	3.76 (1.55–9.13)
SFA										
High					1.00 (Reference)					
Low	0.07	0.33	0.21	0.836	1.07 (0.56–2.06)					
VFA										
High					1.00 (Reference)					
Low	−0.52	0.34	−1.54	0.124	0.59 (0.30–1.15)					
VSR										
High					1.00 (Reference)					
Low	0.02	0.33	0.05	0.962	1.02 (0.53–1.96)					
WBC (×10^9^/L)	0.00	0.03	0.08	0.933	1.00 (0.95–1.06)					
RBC (×10^9^/L)	−0.35	0.21	−1.66	0.097	0.70 (0.46–1.07)					
Hb(g/L)	−0.01	0.01	−1.73	0.083	0.99 (0.98–1.00)					
Plt (×10^9^/L)	−0.00	0.00	−0.83	0.404	1.00 (1.00–1.00)					
NLR	0.01	0.02	0.46	0.647	1.01 (0.97–1.05)					
PLR	0.00	0.00	0.50	0.617	1.00 (1.00–1.00)					
SII	−0.00	0.00	−0.51	0.611	1.00 (1.00–1.00)					
PNI	−0.00	0.01	−0.38	0.703	1.00 (0.98–1.02)					
ALB(g/L)	−0.14	0.03	−4.90	**<0.001**	0.87 (0.82–0.92)	−0.11	0.03	−3.27	**0.001**	0.90 (0.84–0.96)
D-dimer(mg/L)	0.33	0.11	3.01	**0.003**	1.39 (1.12–1.73)					
PT(seconds)	0.20	0.07	2.95	**0.003**	1.23 (1.07–1.40)	0.12	0.08	1.66	0.098	1.13 (0.98–1.31)
FIB(g/L)	0.07	0.11	0.63	0.532	1.07 (0.87–1.32)					
APTT(seconds)	−0.01	0.04	−0.26	0.797	0.99 (0.92–1.06)					

OR, Odds Ratio; CI, Confidence Interval. The bold values represent *p* ≤ 0.05.

**Figure 4:**
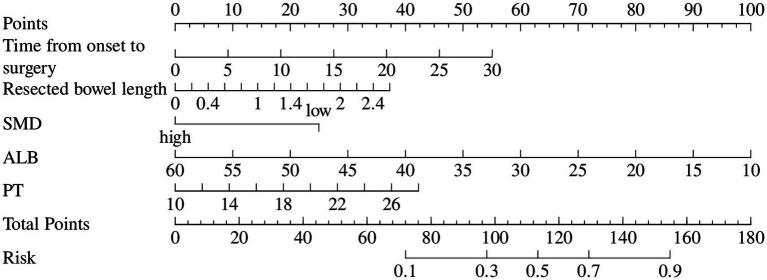
Nomogram for predicting ICU admission.

In the multivariable analysis, five independent predictors for ICU admission were identified. These included longer time from onset to surgery (aOR 1.10, 95% CI 1.02–1.19, *p* = 0.014), greater resected bowel length (aOR 2.14, 95% CI 1.06–4.33, *p* = 0.034), low SMD (aOR 3.76, 95% CI 1.55–9.13, *p* = 0.003), and lower ALB (aOR 0.90, 95% CI 0.84–0.96, *p* = 0.001). Prolonged PT showed a trend toward significance (aOR 1.13, 95% CI 0.98–1.31, *p* = 0.098). Among these independent predictors, low SMD demonstrated the strongest effect size in the final model. The corresponding nomogram is shown in Figure 4.

**Figure 5:**
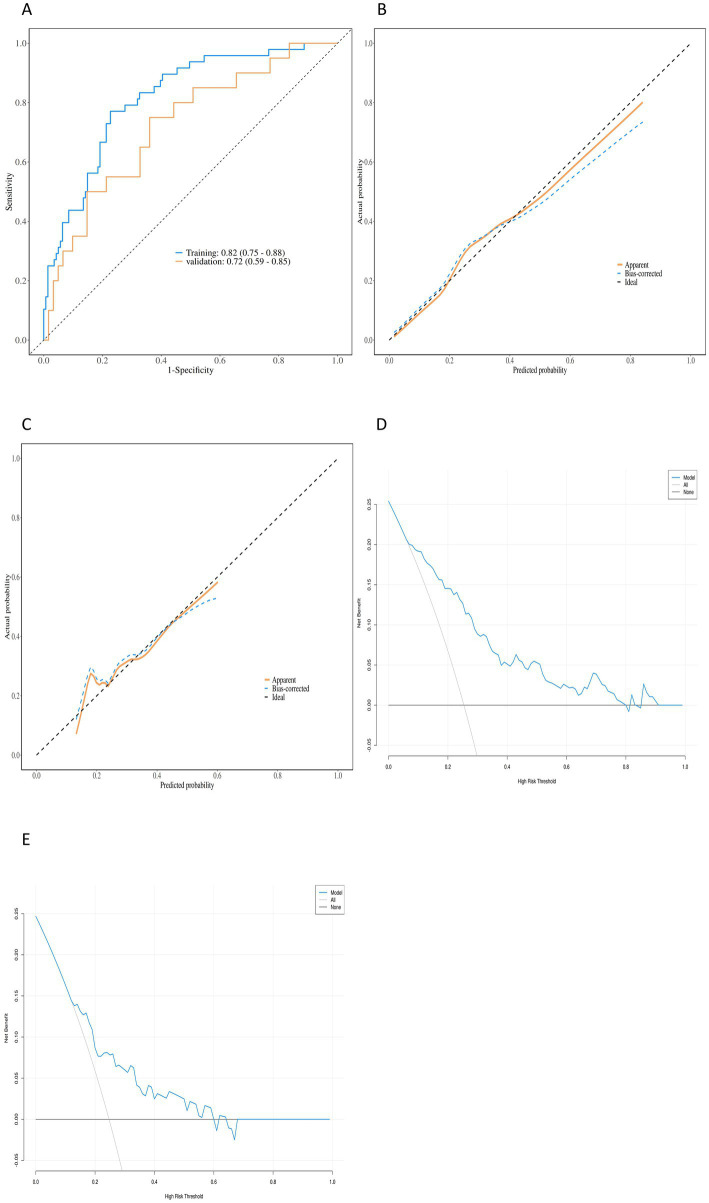
**(A)** ROC curves for the ICU admission prediction model in training and validation sets; **(B)** Calibration curves for the ICU admission prediction model in training sets; **(C)** Calibration curves for the ICU admission prediction model in validation sets; **(D)** Decision curve analysis for the ICU admission prediction model in training sets; **(E)** Decision curve analysis for the ICU admission prediction model in validation sets.

Validation of the model revealed excellent discriminative ability, with an AUC of 0.82 (95% CI: 0.75–0.88) in the training set and 0.72 (95% CI: 0.59–0.85) in the validation set (Figure 5A). The model was well-calibrated (Figures 5B,C), and its clinical value was substantiated by decision curve analysis (Figures 5D,E).

### 3.4 Predictors of postoperative complications

A comparison of demographic and clinical variables between patients with and without clinically significant postoperative complications is provided in Table 5. Table 6 enumerates the independent predictors identified through multivariable analysis for this endpoint. Univariate analysis revealed multiple factors significantly associated with postoperative complications. Advanced age (OR 1.04, 95% CI 1.01–1.06, *p* = 0.002), longer time from onset to surgery (OR 1.09, 95% CI 1.02–1.16, *p* = 0.010), presence of small intestinal necrosis (OR 0.38, 95% CI 0.19–0.77, *p* = 0.007), greater resected bowel length (OR 2.38, 95% CI 1.34–4.26, *p* = 0.003), longer duration of surgery (OR 1.01, 95% CI 1.01–1.01, *p* = 0.007), low SMI (OR 2.54, 95% CI 1.39–4.66, *p* = 0.003), and low SMD (OR 3.61, 95% CI 1.91–6.84, *p* < 0.001) all demonstrated significant associations. Laboratory parameters including lower red blood cell count (OR 0.58, 95% CI 0.39–0.85, *p* = 0.006), lower hemoglobin levels (OR 0.98, 95% CI 0.96–0.99, *p* < 0.001), lower albumin levels (ALB; OR 0.87, 95% CI 0.82–0.91, *p* < 0.001), elevated D-dimer (OR 1.44, 95% CI 1.14–1.82, *p* = 0.002), and prolonged prothrombin time (PT; OR 1.30, 95% CI 1.13–1.49, *p* < 0.001) were also significantly associated with complications. For continuous parameter associations, see Supplementary Table S4.

**Table 5:** Comparison of clinical characteristics between patients with and without postoperative complications.

Variables	No postoperative complications (*n* = 165)	Postoperative complications (*n* = 105)	Statistic	*p*-value
Age, Mean ± SD	60.12 ± 15.83	67.14 ± 13.82	t = −3.73	<0.001
Sex, n(%)			χ^2^ = 0.00	0.978
Female	82 (49.70)	52 (49.52)		
Male	83 (50.30)	53 (50.48)		
BMI, Mean ± SD	21.74 ± 3.36	21.88 ± 5.42	t = −0.23	0.818
Hypertension, n(%)			χ^2^ = 0.72	0.397
No	124 (75.15)	74 (70.48)		
Yes	41 (24.85)	31 (29.52)		
Heart disease, n(%)			χ^2^ = 0.84	0.358
No	153 (92.73)	94 (89.52)		
Yes	12 (7.27)	11 (10.48)		
Diabetes, n(%)			χ^2^ = 1.40	0.237
No	145 (87.88)	97 (92.38)		
Yes	20 (12.12)	8 (7.62)		
Brain disease, n(%)			χ^2^ = 4.01	0.045
No	158 (95.76)	94 (89.52)		
Yes	7 (4.24)	11 (10.48)		
Time from onset to surgery(d), M (Q₁, Q₃)	3.00 (1.00, 4.00)	5.00 (3.00, 8.00)	Z = −4.84	<0.001
Small intestinal necrosis, n(%)			χ^2^ = 11.67	<0.001
Yes	101 (61.21)	85 (80.95)		
No	64 (38.79)	20 (19.05)		
Resected bowel length(m), M (Q₁, Q₃)	0.10 (0.00, 0.30)	0.20 (0.10, 0.80)	Z = −4.39	<0.001
Duration of surgery(min), M (Q₁, Q₃)	110.00 (86.00, 140.00)	125.00 (104.00, 180.00)	Z = −4.26	<0.001
SMI, n(%)			χ^2^ = 6.87	0.009
High	93 (56.36)	42 (40.00)		
Low	72 (43.64)	63 (60.00)		
SMD, n(%)			χ^2^ = 21.34	<0.001
High	101 (61.21)	34 (32.38)		
Low	64 (38.79)	71 (67.62)		
SFA, n(%)			χ^2^ = 0.14	0.708
High	81 (49.09)	54 (51.43)		
Low	84 (50.91)	51 (48.57)		
VFA, n(%)			χ^2^ = 0.76	0.382
High	79 (47.88)	56 (53.33)		
Low	86 (52.12)	49 (46.67)		
WBC(×10^9^/L), M (Q₁, Q₃)	9.40 (6.60, 12.10)	8.60 (6.20, 12.20)	Z = −1.06	0.287
RBC(×10^9^/L), M (Q₁, Q₃)	4.49 (4.12, 5.02)	4.21 (3.68, 4.97)	Z = −2.46	0.014
Hb(g/L), M (Q₁, Q₃)	139.00 (125.00, 154.00)	131.00 (113.00, 148.00)	Z = −2.83	0.005
Plt(×10^9^/L), M (Q₁, Q₃)	233.00 (180.00, 297.00)	199.00 (150.00, 273.00)	Z = −2.30	0.021
NLR, M (Q₁, Q₃)	7.64 (4.29, 13.53)	6.95 (4.86, 16.13)	Z = −0.80	0.425
PLR, M (Q₁, Q₃)	228.09 (156.76, 337.50)	264.06 (174.77, 402.86)	Z = −1.25	0.210
SII, M (Q₁, Q₃)	1740.33 (895.94, 3364.20)	1459.38 (1007.78, 3286.42)	Z = −0.34	0.731
PNI, M (Q₁, Q₃)	45.90 (40.90, 50.00)	38.60 (33.00, 43.50)	Z = −6.54	<0.001
ALB(g/L), M (Q₁, Q₃)	40.10 (35.80, 43.90)	34.00 (28.90, 38.10)	Z = −6.40	<0.001
D-dimer(mg/L), M (Q₁, Q₃)	0.43 (0.27, 0.98)	1.10 (0.58, 2.36)	Z = −6.27	<0.001
PT(seconds), M (Q₁, Q₃)	14.60 (13.70, 15.50)	15.00 (13.50, 17.10)	Z = −2.49	0.013
FIB(g/L), M (Q₁, Q₃)	3.30 (2.78, 4.12)	3.69 (2.80, 4.52)	Z = −1.57	0.117
APTT(seconds), M (Q₁, Q₃)	29.50 (27.50, 32.30)	29.60 (27.10, 31.60)	Z = −0.93	0.351

**Table 6:** Univariate and multivariate logistic regression analysis for predictors of postoperative complications.

Variables	Univariate analysis					Multivariate analysis				
	β	S. E	Z	*p*	OR (95%CI)	β	S. E	Z	*p*	OR (95%CI)
Age	0.04	0.01	3.16	**0.002**	1.04 (1.01–1.06)					
Sex										
Female					1.00 (Reference)					
Male	−0.07	0.30	−0.24	0.808	0.93 (0.52–1.67)					
BMI	−0.03	0.04	−0.77	0.444	0.97 (0.91–1.04)					
Hypertension										
No					1.00 (Reference)					
Yes	−0.16	0.34	−0.48	0.630	0.85 (0.43–1.66)					
Heart disease										
No					1.00 (Reference)					
Yes	0.29	0.50	0.58	0.561	1.34 (0.50–3.56)					
Diabetes										
No					1.00 (Reference)					
Yes	−0.68	0.54	−1.26	0.209	0.51 (0.18–1.46)					
Brain disease										
No					1.00 (Reference)					
Yes	0.94	0.66	1.42	0.155	2.57 (0.70–9.43)					
Time from onset to surgery(d)	0.09	0.03	2.59	**0.010**	1.09 (1.02–1.16)	0.13	0.04	3.26	**0.001**	1.14 (1.05–1.24)
Small intestinal necrosis										
Yes					1.00 (Reference)					
No	−0.97	0.36	−2.68	**0.007**	0.38 (0.19–0.77)					
Resected bowel length(m)	0.87	0.30	2.94	**0.003**	2.38 (1.34–4.26)	0.88	0.36	2.42	**0.016**	2.40 (1.18–4.88)
Duration of surgery(min)	0.01	0.00	2.70	**0.007**	1.01 (1.01–1.01)	0.01	0.00	2.79	**0.005**	1.01 (1.01–1.02)
SMI										
High					1.00 (Reference)					1.00 (Reference)
Low	0.93	0.31	3.02	**0.003**	2.54 (1.39–4.66)	0.74	0.40	1.85	0.064	2.09 (0.96–4.55)
SMD										
High					1.00 (Reference)					1.00 (Reference)
Low	1.28	0.33	3.94	**<0.001**	3.61 (1.91–6.84)	1.32	0.43	3.04	**0.002**	3.73 (1.59–8.71)
SFA										
High					1.00 (Reference)					
Low	0.13	0.30	0.43	0.669	1.14 (0.63–2.05)					
VFA										
High					1.00 (Reference)					
Low	−0.04	0.30	−0.13	0.898	0.96 (0.53–1.73)					
VSR										
High					1.00 (Reference)					
Low	−0.12	0.30	−0.39	0.700	0.89 (0.49–1.60)					
WBC(×10^9^/L)	−0.02	0.03	−0.80	0.425	0.98 (0.93–1.03)					
RBC(×10^9^/L)	−0.55	0.20	−2.77	**0.006**	0.58 (0.39–0.85)					
Hb(g/L)	−0.02	0.01	−3.41	**<0.001**	0.98 (0.96–0.99)					
Plt(×10^9^/L)	−0.00	0.00	−1.76	0.079	1.00 (0.99–1.00)					
NLR	0.01	0.02	0.32	0.752	1.01 (0.97–1.04)					
PLR	0.00	0.00	0.60	0.549	1.00 (1.00–1.00)					
SII	−0.00	0.00	−1.09	0.275	1.00 (1.00–1.00)					
PNI	−0.02	0.02	−1.03	0.304	0.98 (0.95–1.02)					
ALB(g/L)	−0.14	0.03	−5.24	**<0.001**	0.87 (0.82–0.91)	−0.09	0.03	−2.82	**0.005**	0.92 (0.86–0.97)
D-dimer(mg/L)	0.37	0.12	3.04	**0.002**	1.44 (1.14–1.82)					
PT(seconds)	0.26	0.07	3.61	**<0.001**	1.30 (1.13–1.49)	0.22	0.08	2.59	**0.010**	1.24 (1.05–1.46)
FIB(g/L)	−0.06	0.10	−0.58	0.563	0.94 (0.77–1.15)					
APTT(seconds)	0.03	0.03	1.01	0.312	1.03 (0.97–1.10)					

OR, Odds Ratio; CI, Confidence Interval. The bold values represent *p* ≤ 0.05.

**Figure 6:**
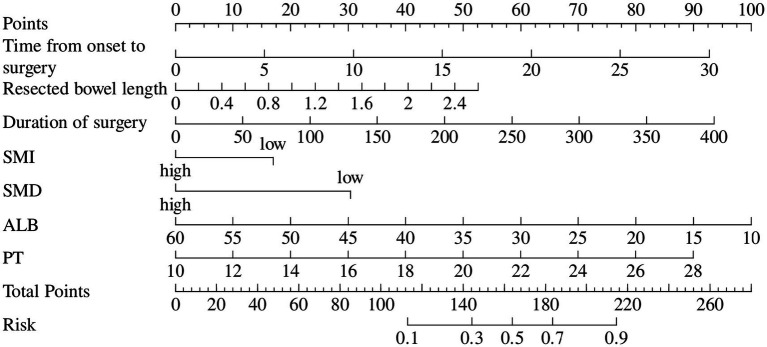
Nomogram for predicting postoperative complications.

In the multivariable analysis, six independent predictors for postoperative complications were identified. These included longer time from onset to surgery (aOR 1.14, 95% CI 1.05–1.24, *p* = 0.001), greater resected bowel length (aOR 2.40, 95% CI 1.18–4.88, *p* = 0.016), longer duration of surgery (aOR 1.01, 95% CI 1.01–1.02, *p* = 0.005), low SMD (aOR 3.73, 95% CI 1.59–8.71, *p* = 0.002), lower ALB (aOR 0.92, 95% CI 0.86–0.97, *p* = 0.005), and prolonged PT (aOR 1.24, 95% CI 1.05–1.46, *p* = 0.010). Low SMI showed a trend toward significance but did not reach statistical significance in the multivariable model (aOR 2.09, 95% CI 0.96–4.55, *p* = 0.064). The comprehensive nomogram is depicted in Figure 6.

**Figure 7:**
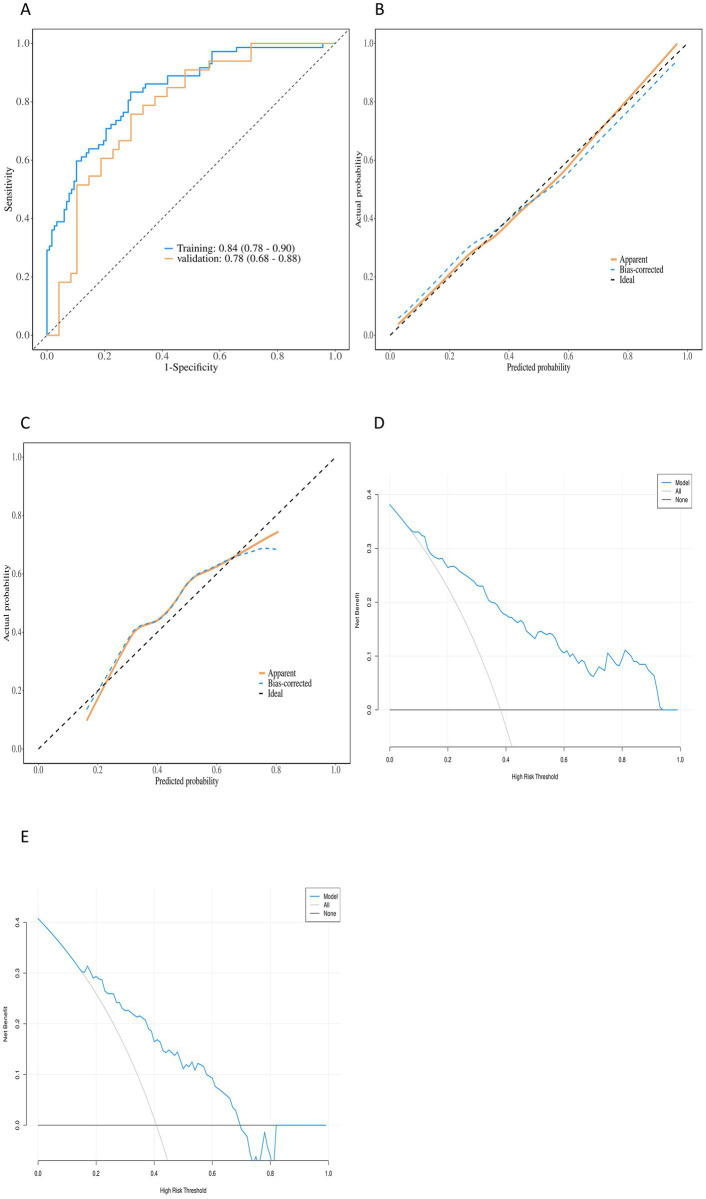
**(A)** ROC curves for the postoperative complications prediction model in training and validation sets; **(B)** Calibration curves for the postoperative complications prediction model in training sets; **(C)** Calibration curves for the postoperative complications prediction model in validation sets; **(D)** Decision curve analysis for the postoperative complications prediction model in training sets; **(E)** Decision curve analysis for the postoperative complications model in validation sets.

The model exhibited robust predictive performance. The AUC reached 0.84 (95% CI: 0.78–0.90) in the training cohort and was maintained at 0.78 (95% CI: 0.68–0.88) upon validation (Figure 7A). Calibration was satisfactory in both cohorts (Figures 7B,C). The decision curve analysis (Figures 7D,E) indicated that applying this model for risk stratification provided a greater net benefit than default strategies across most threshold probabilities.

## 4 Discussion

This study successfully developed and validated three CT-based body composition models for predicting the risk of postoperative sepsis, ICU admission, and clinically significant postoperative complications in patients undergoing surgery for SBO. Independent predictors identified for all adverse outcomes included low SMD, prolonged time from symptom onset to surgery, longer operative time, lower serum albumin levels, elevated D-dimer, prolonged PT, and greater length of resected bowel.

Low SMD, a radiographic marker reflecting muscle fat infiltration, emerged as the most consistent and strongest independent predictor across all three adverse outcomes. This underscores the critical importance of muscle quality in determining physiological resilience when facing the dual assault of acute bowel obstruction and subsequent surgical stress. Pathophysiologically, skeletal muscle fat infiltration may impact outcomes through several pathways: it can reduce muscle contractile function and metabolic activity, impairing respiratory function and mobility (26, 27); furthermore, pro-inflammatory cytokines secreted by adipose tissue may exacerbate systemic inflammation, potentially creating a synergistic effect with the inflammatory state inherent to SBO (28, 29). Additionally, diminished muscle mass compromises protein reserves and immune function, increasing infection risk (30–32). Although previous studies, particularly in elective gastrointestinal surgery populations, often report SMI as a predictor of postoperative complications (33–35), SMI did not remain an independent predictor in our multivariate analysis, with its effect attenuated in multivariable models (for postoperative complications, aOR 2.09, 95% CI 0.96–4.55, *p* = 0.064). We hypothesize that under the intense stress of acute SBO, SMD, reflecting muscle quality, might hold greater prognostic value than SMI, which primarily reflects muscle quantity. Concurrently, intraoperative factors included in this study (such as operative time and length of bowel resection) might partially mediate the risk signaled by low muscle quantity.

The prognostic value of low SMD observed in our SBO cohort aligns with findings from other emergency abdominal surgery settings. For instance, Schwartner et al. (11) reported that low skeletal muscle density independently predicted mortality in acute mesenteric ischemia, while Zhu et al. (13) demonstrated similar associations in upper gastrointestinal perforation. However, the magnitude of effect appears particularly pronounced in SBO (aOR range 3.73–4.58 across outcomes), potentially due to the unique pathophysiology of bacterial translocation and systemic inflammation in obstructed bowel. These comparisons suggest that myosteatosis may serve as a universal marker of physiological frailty across diverse emergency general surgery conditions, though condition-specific thresholds may be warranted.

Although prior literature suggests VFA is associated with risks of infectious complications like postoperative sepsis, potentially via secretion of pro-inflammatory adipokines exacerbating systemic inflammation (36–38), VFA was not an independent predictor in our multivariable analysis. Low VFA showed a protective effect in univariate analysis (OR 0.44), but this effect disappeared after adjusting for other factors (aOR 0.53, *p* = 0.151). One possible explanation lies in the unique pathophysiological changes of acute SBO interfering with VFA measurement. In SBO, proximal bowel distension due to fluid and gas accumulation can compress, displace intra-abdominal fat, and alter its distribution and apparent density on CT cross-sectional images. Moreover, dilated bowel loops themselves might be partially volume-averaged and calculated as intra-abdominal fat, reducing the accuracy of VFA quantification at the L3 level in acute obstruction. Consequently, the reliability of VFA as a risk prediction marker may be compromised in acute bowel obstruction, warranting exploration of optimized measurement techniques or adjusted thresholds in future studies.

Beyond body composition, our models confirmed the key prognostic roles of systemic nutritional status and coagulation profile. Both low SMD and low ALB were significant independent predictors, and while both relate to nutritional status, they likely reflect different physiological mechanisms. ALB, a classic nutritional marker, primarily reflects hepatic synthetic function and protein reserves, influenced by systemic inflammation (IL-6 suppression of synthesis) and fluid balance (39, 40). In contrast, low SMD (sarcopenic obesity) more specifically indicates decreased skeletal muscle quality with fat infiltration, closely linked to insulin resistance, impaired mitochondrial function, and altered basal metabolic rate (28, 29). Thus, ALB may represent current nutritional and inflammatory homeostasis, whereas SMD potentially reveals long-term metabolic health and physiological reserve. Their concurrent and independent significance in the model suggests that risk assessment in SBO patients requires integrating information from both circulating protein levels and muscle quality dimensions. Furthermore, elevated D-dimer was an independent predictor for postoperative sepsis, and prolonged PT was significantly associated with postoperative complications. In SBO, bowel wall edema, ischemia, and tissue necrosis can activate the systemic coagulation system. Elevated D-dimer indicates secondary fibrinolysis and microthrombosis, closely associated with disease severity and adverse outcomes (41–43). Notably, although we systematically evaluated inflammatory cell ratios like NLR, PLR, and SII, none remained independent predictors in the multivariable model. This may indicate that in acute SBO, a potent inflammatory stressor, systemic inflammatory responses are universally and markedly elevated, limiting the ability of these indices to discriminate patients with different prognosis risks. In contrast, markers of coagulopathy (D-dimer, PT) and tissue-specific markers (SMD) exhibited better inter-patient variability, providing more discriminative prognostic information.

Our finding that low SMD independently predicts ICU admission (aOR 3.76, 95% CI 1.55–9.13, *p* = 0.003) adds a novel dimension to existing literature on ICU risk stratification in SBO. Teke and Besler previously investigated predictive markers for ICU requirements in adhesive SBO patients managed nonoperatively, identifying that the number of days since the last stool discharge, Sequential Organ Failure Assessment (SOFA) score, and Charlson Comorbidity Index (CCI) score were significantly associated with ICU need (44). While their study focused on scoring systems and clinical parameters in conservatively managed patients, our study extends these findings in two ways. First, we specifically examined surgically treated SBO patients, a population at higher risk for adverse outcomes. Second, our results suggest that muscle quality may offer complementary prognostic information to acute clinical scores, as it remained independently associated with ICU admission after multivariable adjustment. Notably, in our multivariable model, hypoalbuminemia also emerged as an independent predictor of ICU admission—a finding consistent with Teke and Besler, who reported significantly lower serum albumin levels in their ICU group. Collectively, these results suggest that an optimal ICU triage tool for SBO patients might integrate multiple domains, including acute physiological assessment (SOFA), comorbidity burden (CCI), nutritional reserve (albumin), and muscle quality (SMD). Future prospective studies are needed to compare the performance of models incorporating body composition parameters against traditional scoring systems for predicting ICU resource utilization. Prolonged time from symptom onset to surgery reaffirms the known risk of delayed intervention, allowing progression toward bowel ischemia and bacterial translocation. Longer operative time and greater resection length directly represent surgical complexity and the extent of bowel necrosis, respectively. The latter not only signifies greater disease burden but also portends potential long-term consequences related to malabsorption due to reduced functional bowel length (45, 46).

The final prediction models demonstrated robust performance in both training and validation sets, confirming their generalizability and potential for clinical integration. A practical advantage lies in utilizing routinely available pre- and intra-operative data, facilitating early identification of high-risk individuals. This can inform prehabilitation discussions, guide postoperative monitoring, and optimize ICU resource allocation. Although the analysis utilizes pre-existing CT images and therefore adds no extra radiation exposure or examination cost to the patient, it should be noted that manual or AI-assisted body composition segmentation may incur additional labor or software expenses.

Several limitations warrant consideration. The single-center, retrospective design carries an inherent risk of selection bias. Although the sample size was sufficient for the primary analyses and model development, it limited more extensive subgroup explorations and may affect the stability of estimates for some predictors. Body composition analysis, although meticulously performed by a single researcher with demonstrated high reproducibility, lacks assessment of inter-observer variability across multiple centers. We dichotomized continuous body composition variables (SMD, SMI, VFA) using cohort-specific medians. This approach reduces statistical power and may limit generalizability to other populations. Moreover, body composition indices were dichotomized using cohort-specific medians due to the absence of validated acute SBO thresholds; future studies should aim to establish externally validated cutoffs. The optimal cutoffs for defining low SMD in SBO patients remain to be determined in future external validation studies. Readers should interpret the binary risk estimates as internally valid but not directly transferable to cohorts with different baseline characteristics. Additionally, the model incorporates intraoperative variables, restricting its application to the immediate postoperative phase; future work should develop preoperative-only models for earlier decision support. Finally, the hypothesis regarding potentially inaccurate VFA measurement in the context of bowel obstruction remains a technical consideration. Given the robust internal validation results (AUC 0.78–0.84), we are encouraged by the model’s potential, yet we recognize that external validation in independent cohorts from diverse geographic and healthcare settings is a necessary next step before clinical translation. The observed complication rate (38.9%) is comparable to those reported in large European and North American series, which suggests potential applicability in external populations; however, multi-center, prospective external validation is required before any clinical extrapolation (6, 9). Future research should prioritize external validation in multi-center, prospective cohorts; investigate integrated AI-based automated body composition tools to enhance reproducibility and accuracy; and further explore the mechanistic interplay between sarcopenic obesity, coagulopathy, and outcomes in SBO.

## 5 Conclusion

This study establishes low SMD, a marker of myosteatosis, as a powerful and independent predictor of major adverse outcomes following SBO surgery. It operates alongside, yet distinct from, the pathway represented by systemic inflammation and nutritional status (ALB). When combined with key surgical factors, these variables provide a comprehensive framework for individual risk stratification. Our results advocate for incorporating body composition analysis into early postoperative risk assessment, moving beyond traditional metrics to utilize data readily available from standard CT scans, thereby enabling more personalized postoperative management strategies.

## Data Availability

The raw data supporting the conclusions of this article will be made available by the authors, without undue reservation.
