# Supplementary material for: Development and validation of a CT-based body composition model for predicting adverse outcomes in small bowel obstruction
Source: Front Med (Lausanne). 2026 Jun 1;13:1807639. doi: 10.3389/fmed.2026.1807639 (PMC13265353; doi:10.3389/fmed.2026.1807639)
Supplement: Supplementary file 1 [file Data_Sheet_1.DOCX]

## **Table S1. Inter‑rater reliability of body composition measurements (n = 60)**

| Parameter | ICC | 95% CI |
| --- | --- | --- |
| SMD (HU)  SMD（HU） | 0.95 | 0.91 – 0.97 |
| SMI | 0.98 | 0.96 – 0.99 |
| VFA | 0.96 | 0.91 – 0.98 |
| SFA | 0.92 | 0.87 – 0.96 |

ICC: intraclass correlation coefficient (two‑way random, absolute agreement); SMD: skeletal muscle density; SMI: skeletal muscle index; VFA: visceral fat area; SFA: subcutaneous fat area. Measurements were independently performed by two investigators blinded to outcomes on a randomly selected subset of 60 patients (~22% of cohort).

## **Table S2. Temporal distribution and trend analysis of main outcomes across the study period (2018–2024)**

| Year | Total N | Postoperative sepsis, n (%) | ICU admission, n (%) | CD ≥ II complications, n (%) |
| --- | --- | --- | --- | --- |
| 2018 | 30 | 6 (20.0) | 7 (23.3) | 12 (40.0) |
| 2019 | 35 | 7 (20.0) | 8 (22.9) | 13 (37.1) |
| 2020 | 38 | 7 (18.4) | 10 (26.3) | 15 (39.5) |
| 2021 | 42 | 9 (21.4) | 11 (26.2) | 16 (38.1) |
| 2022 | 45 | 9 (20.0) | 12 (26.7) | 18 (40.0) |
| 2023 | 42 | 8 (19.0) | 11 (26.2) | 17 (40.5) |
| 2024 | 38 | 7 (18.4) | 9 (23.7) | 14 (36.8) |
| P for trend | — | 0.89 | 0.76 | 0.92 |

**Table S3. Comparison of baseline demographic and clinical characteristics between the training (n=189) and validation (n=81) cohorts.**

| Variables | Total (n = 270) | test (n = 81) | train (n = 189) | Statistic | *P* |
| --- | --- | --- | --- | --- | --- |
|  |  |  |  |  |  |
| Age, Mean ± SD | 62.85 ± 15.44 | 63.22 ± 14.92 | 62.69 ± 15.69 | t=0.26 | 0.797 |
| Sex, n(%) |  |  |  | χ²=0.05 | 0.832 |
| Female | 134 (49.63) | 41 (50.62) | 93 (49.21) |  |  |
| Male | 136 (50.37) | 40 (49.38) | 96 (50.79) |  |  |
| BMI, Mean ± SD | 21.80 ± 4.27 | 21.60 ± 3.32 | 21.88 ± 4.62 | t=-0.50 | 0.616 |
| Hypertension, n(%) |  |  |  | χ²=1.75 | 0.186 |
| No | 198 (73.33) | 55 (67.90) | 143 (75.66) |  |  |
| Yes | 72 (26.67) | 26 (32.10) | 46 (24.34) |  |  |
| Heart disease, n(%) |  |  |  | χ²=0.18 | 0.669 |
| No | 247 (91.48) | 75 (92.59) | 172 (91.01) |  |  |
| Yes | 23 (8.52) | 6 (7.41) | 17 (8.99) |  |  |
| Diabetes, n(%) |  |  |  | χ²=1.28 | 0.257 |
| No | 242 (89.63) | 70 (86.42) | 172 (91.01) |  |  |
| Yes | 28 (10.37) | 11 (13.58) | 17 (8.99) |  |  |
| Brain Disease, n(%) |  |  |  | χ²=1.63 | 0.201 |
| No | 252 (93.33) | 78 (96.30) | 174 (92.06) |  |  |
| Yes | 18 (6.67) | 3 (3.70) | 15 (7.94) |  |  |
| Time from onset to surgery(d), M (Q₁, Q₃) | 3.00 (2.00, 6.00) | 3.00 (2.00, 6.00) | 3.00 (2.00, 7.00) | Z=-0.84 | 0.399 |
| Small intestinal necrosis, n(%) |  |  |  | χ²=0.27 | 0.606 |
| Yes | 186 (68.89) | 54 (66.67) | 132 (69.84) |  |  |
| No | 84 (31.11) | 27 (33.33) | 57 (30.16) |  |  |
| Resected bowel length(m), M (Q₁, Q₃) | 0.10 (0.00, 0.50) | 0.15 (0.00, 0.50) | 0.10 (0.00, 0.50) | Z=-0.06 | 0.953 |
| Duration of surgery(min), M (Q₁, Q₃) | 120.00 (90.00, 150.00) | 120.00 (92.00, 155.00) | 120.00 (90.00, 150.00) | Z=-0.39 | 0.695 |
| SMI, n(%) |  |  |  | χ²=0.44 | 0.507 |
| high | 135 (50.00) | 38 (46.91) | 97 (51.32) |  |  |
| low | 135 (50.00) | 43 (53.09) | 92 (48.68) |  |  |
| SMD, n(%) |  |  |  | χ²=0.02 | 0.894 |
| high | 135 (50.00) | 40 (49.38) | 95 (50.26) |  |  |
| low | 135 (50.00) | 41 (50.62) | 94 (49.74) |  |  |
| SFA, n(%) |  |  |  | χ²=0.16 | 0.690 |
| high | 135 (50.00) | 42 (51.85) | 93 (49.21) |  |  |
| low | 135 (50.00) | 39 (48.15) | 96 (50.79) |  |  |
| VFA, n(%) |  |  |  | χ²=1.43 | 0.232 |
| high | 135 (50.00) | 45 (55.56) | 90 (47.62) |  |  |
| low | 135 (50.00) | 36 (44.44) | 99 (52.38) |  |  |
| VSR, n(%) |  |  |  | χ²=0.86 | 0.353 |
| high | 135 (50.00) | 37 (45.68) | 98 (51.85) |  |  |
| low | 135 (50.00) | 44 (54.32) | 91 (48.15) |  |  |
| WBC(×10⁹/L), M (Q₁, Q₃) | 9.05 (6.32, 12.17) | 8.10 (6.20, 11.90) | 9.30 (6.60, 12.20) | Z=-0.89 | 0.374 |
| RBC(×10⁹/L), M (Q₁, Q₃) | 4.39 (3.90, 5.01) | 4.26 (3.82, 5.04) | 4.41 (3.92, 4.99) | Z=-0.92 | 0.356 |
| Hb(g/L), M (Q₁, Q₃) | 136.00 (121.00, 153.00) | 134.00 (121.00, 153.00) | 137.00 (121.00, 151.00) | Z=-0.32 | 0.747 |
| Plt(×10⁹/L), M (Q₁, Q₃) | 222.00 (172.00, 287.75) | 230.00 (178.00, 286.00) | 219.00 (170.00, 288.00) | Z=-0.09 | 0.926 |
| NLR, M (Q₁, Q₃) | 7.36 (4.55, 14.19) | 6.79 (4.18, 11.96) | 7.64 (4.77, 15.71) | Z=-1.11 | 0.267 |
| PLR, M (Q₁, Q₃) | 244.25 (165.63, 365.75) | 247.42 (156.76, 332.56) | 235.38 (167.71, 404.55) | Z=-0.98 | 0.328 |
| SII, M (Q₁, Q₃) | 1559.79 (971.09, 3344.75) | 1427.29 (879.38, 2609.27) | 1678.56 (976.11, 3589.00) | Z=-1.10 | 0.272 |
| PNI, M (Q₁, Q₃) | 43.35 (36.66, 48.34) | 43.35 (38.35, 47.55) | 43.40 (36.50, 48.35) | Z=-0.16 | 0.871 |
| ALB(g/L), M (Q₁, Q₃) | 37.50 (32.32, 42.18) | 37.50 (32.30, 42.40) | 37.80 (32.40, 42.10) | Z=-0.31 | 0.753 |
| D-dimer(mg/L), M (Q₁, Q₃) | 0.63 (0.32, 1.34) | 0.57 (0.29, 1.17) | 0.71 (0.33, 1.44) | Z=-0.65 | 0.517 |
| PT(seconds), M (Q₁, Q₃) | 14.70 (13.62, 16.08) | 14.60 (13.70, 15.80) | 14.80 (13.60, 16.20) | Z=-0.72 | 0.474 |
| FIB(g/L), M (Q₁, Q₃) | 3.43 (2.80, 4.33) | 3.50 (2.88, 4.33) | 3.40 (2.75, 4.29) | Z=-0.90 | 0.368 |
| APTT(seconds), M (Q₁, Q₃) | 29.55 (27.42, 31.98) | 29.80 (27.60, 32.30) | 29.30 (27.40, 31.80) | Z=-0.32 | 0.751 |
| t: t-test, Z: Mann-Whitney test, χ²: Chi-square test | | | | | |
| SD: standard deviation, M: Median, Q₁: 1st Quartile, Q₃: 3st Quartile | | | | | |

## **Table S4. Univariate associations of body composition parameters as continuous variables with the three primary outcomes**

| Outcome | Parameter | OR (95% CI) | P value |
| --- | --- | --- | --- |
| Postoperative sepsis | SMD (HU)  SMD（HU） | 0.96 (0.93–0.98) | <0.001 |
|  | SMI | 0.92 (0.87–0.97) | 0.003 |
|  | VFA | 1.02 (0.98–1.06) | 0.32 |
| ICU admission  ICU | SMD (HU)  SMD（HU） | 0.95 (0.92–0.97) | <0.001 |
|  | SMI | 0.90 (0.85–0.95) | <0.001 |
|  | VFA | 1.00 (0.97–1.04) | 0.85 |
| CD ≥ II complications | SMD (HU)  SMD（HU） | 0.96 (0.94–0.98) | <0.001 |
|  | SMI | 0.93 (0.88–0.98) | 0.004 |
|  | VFA | 1.01 (0.98–1.04) | 0.51 |

Abbreviations: SMD, skeletal muscle density (HU); SMI, skeletal muscle index (cm²/m²); VFA, visceral fat area (cm²); OR, odds ratio per 1‑unit increment (for SMD and VFA) or per 1 cm²/m² increment (for SMI).
